# The Turkish version of the Modified Falls Efficacy Scale: reliability and validity from the viewpoint of balance

**DOI:** 10.3906/sag-1903-212

**Published:** 2019-12-16

**Authors:** Nilüfer ÇETİŞLİ KORKMAZ, Mehmet DURAY, Esra DOĞRU HÜZMELİ, Hande ŞENOL

**Affiliations:** 1 School of Physiotherapy and Rehabilitation, Pamukkale University, Denizli Turkey; 2 Faculty of Health Sciences, Department of Physiotherapy and Rehabilitation, Mustafa Kemal University, Hatay Turkey; 3 Department of Biostatistics, Faculty of Medicine, Pamukkale University, Denizli Turkey

**Keywords:** Modified Falls Efficacy Scale, falls, fear of falling, Berg Balance Scale, older people, elderly

## Abstract

**Background/aim:**

The factors associated with fall-related self-efficacy should be addressed, especially in the elderly. The Modified Falls Efficacy Scale (MFES) is a simple instrument with good scaling properties and reliability. The aim of the present study was to determine the reliability and the validity of the Turkish version of the MFES from the viewpoint of balance.

**Materials and methods:**

In this study, 164 participants aged >65 years were included. The use of walking aids and assistive devices, history and frequency of falls in the previous year, living environment, and exercise habits were noted. Balance and risk of falling were assessed with the Berg Balance Scale (BBS). A forward-backward translation procedure was used for the Turkish version of the MFES.

**Results:**

None of the 14 items in the MFES were modified. The Turkish version of the MFES has excellent internal consistency (Cronbach’s alpha, 0.978) and reliability (interclass correlation coefficient, 0.928–0.982), and its construct validity was supported by its ability to distinguish between the groups with respect to fall-risk factors and balance. According to the BBS scores, the high-fall-risk group had lower MFES scores than the moderate- and low-fall-risk groups (χ2 = 34.153, P = 0.001).

**Conclusion:**

The Turkish version of the MFES is a sensitive instrument for evaluation of fall-related confidence while carrying out indoor/outdoor activities. It also predicts falls, reduced physical activity, balance and mobility problems, and restricted social participation and daily living activities.

## 1. Introduction

Falls due to physical and psychological factors, often seen among the elderly, are a major public health concern [1]. The history and risk of falls restrict voluntary activities due to the fear of reoccurrence of falls. The deterioration in health status impairs balance and body function by ultimately reducing the fall-related self-efficacy, defined as the loss of one’s confidence in performing the activities of daily living without falling [2,3]. Psychological consequences of falls, including low fall-related self-efficacy, might be determinants of repeated falls, balance problems, depression level, limitations in activities of daily life, physical inactivity, perceived poor health, loss of functional independence and confidence, and restrictions in social participation. They often result in a decrease in quality of life and adversely increase the socioeconomic burden of society [1,4,5], reportedly causing more disabilities than the falls themselves [6,7]. Therefore, fall-related self-efficacy should be addressed and subsequent intervention strategies should be developed, especially for the elderly.

The risk factors for falls are classified as intrinsic (age-related personal changes) and extrinsic (environmental) risk factors [7]. The intrinsic risk factors, including physiological, biological, anatomical, and psychological changes, e.g., low fall-related self-efficacy with aging, are accepted as the primary cause of falls [8–11]. Therefore, the relation between fall-related self-efficacy and the other risk factors for falls should be considered to assess and improve the fall-related self-efficacy level in the elderly that are participating in physiotherapy and rehabilitation programs.

The Falls Efficacy Scale (FES) is the most commonly used and the best-established questionnaire that measures confidence related to falls. The FES aims to measure self-perceived confidence while performing 10 specific daily activities in the elderly [6,12]. However, the FES might be inadequate to evaluate self-confidence related to the outdoors because it does not adequately evaluate the social dimensions of falling and has cultural incompatibility [12].

Hill et al. expanded the FES to develop a reliable and valid instrument, known as the Modified Falls Efficacy Scale (MFES) [13]. The MFES is a modified version of the original 10-item FES, which could be used in different languages and cultural settings. The MFES includes 4 additional outdoor activity tasks inducing loss of fall-related self-efficacy in the elderly, and it is a clear and simple instrument with good scaling properties and test-retest reliability [14].

The Turkish version of the MFES has not been studied from the viewpoint of balance yet. Therefore, the aim of this study was to develop the Turkish version of the MFES and to determine its reliability and validity in terms of balance in older adults.

## 2. Materials and methods

### 2.1. Participants

A total of 164 participants, aged >65 years, volunteered for the study. Those with a Mini-Mental State Examination (MMSE) score of <17, blindness, deafness, or an acute musculoskeletal complaint were excluded from the study. Informed consent was obtained from all participants, and the study was performed in accordance with the Declaration of Helsinki and was approved by the Ethics Committee for Human Investigations of Mustafa Kemal University (4298783 / 0533).

### 2.2. Study procedure and instruments

The study procedure and all measurements were performed in the same assessment session and the MFES was applied at intervals of 5–7 days for the reliability of the procedure. The MMSE, the Berg Balance Scale (BBS), and the MFES were used for the evaluation.

#### 2.2.1. Sociodemographic data form

A semistructured interview form was devised by the researchers to record the sociodemographic data and clinical information obtained from the participants. The use of assistive devices, history and frequency of falls in the last year, living environment, and exercise habits were also recorded. 

#### 2.2.2. Mini-Mental State Examination

The MMSE was used for the evaluation of cognitive functions. The highest achievable score is 30 and scores below 24 show cognitive impairment in people aged >65 years [15]. 

#### 2.2.3. Berg Balance Scale 

Participants’ risk of falling was measured with the BBS. It consists of 14 items. The total score of the scale is 56 and higher scores show decreased risk of fall [16].

#### 2.2.4. Modified Falls Efficacy Scale 

The MFES is an expanded form of the FES in which the items questioning the confidence during 4 different outdoor activities were included. The 14 items in the MFES (10 indoor and 4 outdoor activities), assess the confidence during performing different daily tasks. The items on the scale are scored from 0 (not confident) to 10 (completely confident) to rate the participants’ fall-related self-efficacy level [13].

#### 2.2.5. Study procedure

The linguistic validity was assessed as described in the literature (Step 1: investigation of conceptual and item equivalence, Step 2: original instrument was translated, Step 3: a synthesized translated version, Step 4: back translations, and Step 5: a synthesized back-translated version) [17]. The permission to translate and culturally adapt the MFES into Turkish was obtained from Hill by the first author of the original instrument. After receiving permission, the original version of the MFES was translated into Turkish independently by 2 Turkish native speakers who were familiar with the concept of fear of falling, and then a single form was prepared by consensus of the translators. Subsequently, the MFES, in a written form, was tested by each of the translators with 2 older people to evaluate its comprehensibility and appropriateness. In a second meeting, which was conducted for consensus on necessary changes, it was decided that no significant adaptations were needed. This Turkish version was then back-translated to English by 2 independent professional translators whose native language was English and were not aware of the intent. Another consensus meeting of the translators was done to review the back-translation. The final version of the back-translated form was then compared with the original MFES by another independent physiotherapist to determine whether semantic and conceptual equivalence was met. A final Turkish version of the MFES was obtained with the completion of all these steps.

### 2.3. Modified Falls Efficacy Scale administration

All data were collected during the participants’ initial visits to the clinic and after intervals of 5–7 days from the first assessment because it was presumed that the clinical status remained unchanged within this interval [18]. The MFES was administered by the physiotherapists under the supervision of 2 experienced physiotherapists who were working in the geriatric rehabilitation unit. The participants were supported when necessary. Any questions regarding the procedure and the MFES were explained to the participants without any commentary.

### 2.4. Statistical analysis

Descriptive statistics were used to describe the characteristics of the participants. All continuous variables were evaluated for normality using the Kolmogorov–Smirnov test. They were expressed as mean ± standard deviation (SD) (if data were normally distributed) or as medians in combination with the quartiles and percentiles (if data were not normally distributed). Relative reliabilities and the internal consistency of the items of the MFES were determined with the interclass correlation coefficient (ICC) and Cronbach’s coefficients, respectively. The MFES scores of the groups, which were classified according to the demographic properties, were compared by one-way analysis of variance, Kruskal–Wallis variance analysis, independent samples t-test, and Mann–Whitney U test. In addition, the correlations between the MFES score and sex, assistive device, living environment, history and frequency of falls, exercise habits, BBS score, and MMSE score were examined by the Spearman correlation (two-tailed) test.

## 3. Results

Out of the total 164 participants aged >65 years (mean age: 74.11 ± 7.9 years) included in the study, 59 were female and 105 were male. Their mean frequency of falling in the previous year was 0.74 ± 1.08, while their BBS score was 42.12 ± 13.44. The means of MFES1 scores (scores obtained from the first assessment) and MFES2 scores (scores obtained from the retest) were 6.28 ± 2.26 and 6.67 ± 2.19, respectively. The demographic and clinical characteristics of the participants are shown as mean ± SD and percentage in Table 1.

**Table 1:** Demographic and clinical characteristics of participants.

			n	%
Sex	Male		105	64.0
	Female		59	36.0
Marital status	Married		49	29.9
	Single		20	12.2
	Widowed		95	57.9
Education level	Literate		60	36.5
	Primary school		48	29.3
	Middle school		20	12.2
	High school		25	15.2
	College/university		11	6.7
Hearing / vision loss	Yes		40	26.7
	No		110	73.3
Disease history	Cardiovascular		51	31.1
	Pulmonary		17	10.4
	Metabolic		25	15.2
	Musculoskeletal system		16	9.8
	No disease		55	33.5
Using assistive devices	Hearing / vision		29	17.7
	Walking assistance (cane, walker…)		16	9.7
	Wheelchair		8	4.9
	Other		5	3.0
	No assistive devices		106	64.6
Living environment	With wife		43	28.7
	With children		21	14.0
	Nursing home		79	52.7
	Alone		7	4.70
Exercise habits	Yes	1–2 days/week	10	6.7
		3–5 days/week	10	6.7
		6–7 days/ week	6	4.0
	No		124	82.7
Falling history	Yes		71	43.3
	No		93	56.7
BBS (Risk of fall)	0–20 (High risk)		16	9.8
	21–40 (Moderate risk)		38	23.2
	41–56 (Low risk)		110	67.1

BBS: Berg Balance Scale.

We found that the Bartlett’s test result was P < 0.0001 and the Kaiser–Meyer–Olkin (KMO) value was 0.948. Therefore, we applied factor analysis as the obtained values showed that the data were appropriate for the analysis (Bartlett’s test result was P < 0.001 and KMO value was ˃0.6). We found that the MFES scale has a one-factor structure with principal component analysis in structural validity and factor analysis. We calculated that 78.28% of the total variance could be explained by this one-factor structure. However, the factor loadings obtained as a result of the principal component analysis indicate that the scale has a one-factor structure and all the items could be used (Table 2).

**Table 2:** Results of internal consistency and test-retest reliability of the Turkish version of the MFES.

	MFES_1_	MFES_2_	Corrected I-TC	CA-iid.	ICC (%95 C.I)	PCA
Item 1	7.01 ± 2.40	7.41 ± 2.22	0.820	0.977	0.977 (0.968–0.983)	0.848
Item 2	6.84 ± 2.55	7.09 ± 2.37	0.841	0.977	0.968 (0.956–0.976)	0.866
Item 3	6.22 ± 2.53	6.59 ± 2.46	0.835	0.977	0.977 (0.969–0.983)	0.860
Item 4	6.74 ± 2.41	7.02 ± 2.31	0.923	0.975	0.979 (0.971–0.984)	0.939
Item 5	6.82 ± 2.44	7.09 ± 2.31	0.925	0.975	0.982 (0.976–0.987)	0.940
Item 6	7.10 ± 2.45	7.37 ± 2.32	0.904	0.976	0.976 (0.968–0.983)	0.922
Item 7	6.96 ± 2.48	7.26 ± 2.41	0.892	0.976	0.966 (0.954–0.975)	0.912
Item 8	5.99 ± 2.50	6.59 ± 2.55	0.872	0.976	0.956 (0.941–0.968)	0.891
Item 9	5.88 ± 2.61	6.42 ± 2.55	0.862	0.976	0.957 (0.942–0.969)	0.880
Item 10	6.14 ± 2.64	6.62 ± 2.59	0.895	0.976	0.966 (0.954–0.975)	0.909
Item 11	5.70 ± 2.67	6.16 ± 2.62	0.866	0.976	0.961 (0.946–0.971)	0.881
Item 12	5.53 ± 2.60	5.98 ± 2.60	0.820	0.977	0.928 (0.903–0.947)	0.841
Item 13	5.23 ± 2.97	5.50 ± 2.84	0.782	0.978	0.936 (0.913–0.953)	0.807
Item 14	5.73 ± 2.52	6.23 ± 2.44	0.860	0.976	0.942 (0.921–0.957)	0.879

CA-iid: Cronbach’s alpha if item deleted, ICC: interclass correlation coefficient, I-TC: item-total correlation, PCA: principal component analysis.

### 3.1. Reliability

To test the reliability of the scale, we determined that the Cronbach alpha value (0.978) of 14 items in the MFES2 score was the same as that obtained from MFES1. The ICC values (0.928–0.982) and Cronbach alpha values of all items were very high for the Turkish version of the MFES. Therefore, these results showed that the internal consistency test of the MFES had high test-retest reliability. We also found that it was not necessary to remove any item from the scale according to the Cronbach alpha values (0.975–0.978) and corrected item-total correlation values (0.782–0.925) obtained when any item was removed. The measurement ability of all items was quite high (Table 2).

We recorded very strong correlations between the items examined. The lowest correlation between items was found between the 1st and the 12th items (r = 0.591), whereas the strongest correlation was between the 4th and the 5th items (r = 0.967) (Table 3).

**Table 3:** Interitem correlation matrix of the MFES.

	1	2	3	4	5	6	7	8	9	10	11	12	13	14
1	1	0.849	0.729	0.818	0.812	0.868	0.791	0.740	0.684	0.701	0.665	0.591	0.602	0.645
2		1	0.750	0.810	0.799	0.847	0.779	0.718	0.752	0.722	0.716	0.663	0.639	0.674
3			1	0.854	0.816	0.793	0.755	0.718	0.688	0.727	0.745	0.742	0.592	0.738
4				1	0.967	0.895	0.880	0.816	0.772	0.810	0.760	0.716	0.673	0.824
5					1	0.888	0.886	0.833	0.79	0.828	0.766	0.724	0.686	0.819
6						1	0.890	0.796	0.755	0.780	0.749	0.688	0.686	0.757
7							1	0.845	0.739	0.830	0.729	0.704	0.657	0.780
8								1	0.818	0.854	0.700	0.710	0.730	0.744
9									1	0.828	0.800	0.721	0.780	0.768
10										1	0.814	0.806	0.758	0.797
11											1	0.865	0.773	0.845
12												1	0.731	0.771
13													1	0.724
14														1

### 3.2. Validity

We found that the average of the MFES1 and MFES2 scores for the entire sample decreased when the risk of fall increased, as shown in Table 4. In both the first and second applications of the MFES, the scores of the participants with a high risk of falling were significantly lower than those of the participants with medium and low risks of falling (P < 0.01). The MFES1 scores were significantly different when divided into separate subgroups according to the demographic properties, such as marital status, hearing problems, medical history, living environment, and history and risk of falling. We found that there were significant differences between the groups (P < 0.05), except sex, dominant side, education level, vision loss, and exercise habits (Table 5). In addition to these, correlations were obtained between the MFES score and history and frequency of falling (r = 0.292, P = 0.000 and r = -0.274, P = 0.000, respectively), and the BBS score (r = -0.465, P = 0.000). 

**Table 4:** Comparison of the test-retest MFES results among the groups according to the risk of falling.

	MFES_1_				MFES_2_			
	X ± SD	χ^2^	P	Post hoc	X ± SD	χ^2^	P	Post hoc
0–20 (High risk) (n = 16) (1)	3.79 ± 2.42	34.153	0.001***	1–3, 1–2	4.58 ± 2.35	34.655	0.001***	1–3, 1–2
21–40 (Moderate risk) (n = 38) (2)	5.20 ± 2.41				5.35 ± 2.40			
41–56 (Low risk) (n = 110) (3)	7.00 ± 1.70				7.43 ± 1.62			

MFES_1_: Modified Falls Efficacy Scale (pretest), MFES_2_: Modified Falls Efficacy Scale (posttest), χ^2^: Kruskal–Wallis variance analysis, *** P ≤ 0.001.

**Table 5:** The comparison of the MFES scores among the groups, according to their demographic characteristics.

		MFES X ± SD (min–max)	Test value	P	Post hoc
Sex	Male (n = 105)	6.44 ± 2.22 (0.93–10)	t = 1.259	0.210	-
	Female (n = 59)	5.98 ± 2.31 (0.64–10)			
Dominance	Right (n = 143)	6.28 ± 2.28 (0.64–10)	t = 0.097	0.923	-
	Left (n = 21)	6.23 ± 2.14 (1.71–9.29)			
Marital status	Married (n = 49) (1)	6.87 ± 2.25 (2.29–10)	F = 3.912	0.022*	1–3
	Single (n = 20) (2)	6.78 ± 2.47 (0.93–9.79)			
	Widowed (n = 95) (3)	5.86 ± 2.15 (0.64–10)			
Education level	Literate (n = 60)	3.26 ± 1.28 (2–6)	F = 0.800	0.551	-
	Primary school (n = 48)	6.18 ± 2.03 (1.57–9.64)			
	Middle school (n = 20)	6.18 ± 2.16 (1.71–9.36)			
	High school (n = 25)	6.36 ± 2.45 (2.07–9.64)			
	College/university (n = 11)	7.29 ± 2.07 (2.86–10)			
Mini-Mental Test	24–30: Normal (n = 72) (1)	7.00 ± 2.14 (0.93–10)	F = 8.111	0.001***	1–21–3
	18–23: Mild dementia (n = 71) (2)	5.88 ± 2.07 (1.71–9.93)			
	<18: High dementia (n = 21) (3)	5.13 ± 2.53 (0.64–9.57)			
Hearing loss	Yes (n = 40)	5.47 ± 2.25 (0.64–9.64)	z = –2.089	0.037*	-
	No (n = 110)	6.15 ± 2.25 (1.71–10)			
Vision loss	Yes (n = 40)	6.41 ± 2.14 (2.07–10)	t = –0.127	0.899	-
	No (n = 110)	6.2 ± 2.07 (0.64–9.43)			
Using assistive devices	Hearing (n = 17) (1)	6.36 ± 2.23 (3.43–9.57)	χ^2^ = 24.139	0.001***	1–4 2–4 6–4
	Vision (n = 12) (2)	6.61 ± 2.23(3.00–9.79)			
	Walking assistance (cane, walker…) (3)	5.87 ± 2.67 (1.71–9.64)			
	Wheelchair (n = 8) (4)	2.93 ± 0.72 (1.57–4)			
	Other (n = 5) (5)	3.58 ± 2.39 (0.93–6.93)			
	No assistive devices (n = 106) (6)	6.65 ± 2.02 (0.64–10)			
Living environment	With spouse (n = 43) (1)	6.81 ± 2.22 (2.29–9.93)	χ^2^ = 12.346	0.006**	1–2
	With children (n = 21) (2)	4.86 ± 1.7 (2.07–8.36)			
	Nursing home (n = 79) (3)	6.19 ± 2.19 (0.64–10)			
	Alone (n = 7) (4)	5.81 ± 2.09 (3.86–10)			
Exercise habit	Yes (n = 26)	6.64 ± 1.79 (3.50–10)	t = 1.427	0.161	-
	No (n=124)	6.06 ± 2.27 (0.64–10)			
Falling history	Yes (n = 71)	5.54 ± 2.11 (0.64–9.93)	t = –3.818	0.001***	-
	No (n = 93)	6.84 ± 2.21 (0.93–10)			

F: One-way analysis of variance, χ^2^: Kruskal–Wallis variance analysis, t: independent samples t-test, z: Mann–Whitney U test, *P < 0.05, **P < 0.01, ***P ≤ 0.001.

## 4. Discussion

This study demonstrated that the MFES has good to excellent reliability, internal consistency, and validity for evaluation of fall efficacy in the elderly. The findings of this study confirmed that the Turkish version of the MFES could be an effective and sensitive instrument to prompt self-confidence evaluations in older people.

The decrease in fall-related self-efficacy significantly contributes to functional decline and deterioration in well-being of the elderly. Therefore, various fall-related efficacy scales have been developed to quantify perceived self-confidence in the elderly [19]. Unlike other fall efficacy scales, the MFES is a multidimensional scale, which was developed to assess efficacy during simple physical outdoor activities along with indoor activities [3,13]. Harding et al. proposed that due to this feature, researchers and clinicians should use the MFES as an alternative instrument instead of the FES, especially for outdoor activities of community-dwelling older people [20]. Different versions of the MFES have been developed in various languages [6,14,21] and were used in rehabilitation research and clinical trials [2,3,22,23]. To the best of our knowledge, no specific Turkish adapted instrument for fall-related self-efficacy including outdoor activities has previously been analyzed from the viewpoint of balance in the elderly.

The stability of the answers for each item represents the test-retest reliability of a scale over time [13,14]. We found that the ICC values of all items (0.928–0.982) and the Cronbach alpha values were very high (ranging from 0.903 to 0.987) in this study, showing the consistency of a measure when used repeatedly and pointing to the excellent reliability of the MFES. When we investigated the ICC values, we noted that the Turkish version of the MFES showed sufficient reproducibility, similar to the original MFES (0.54–0.98) [15]. The ICC values obtained from the test-retest studies for the Serbian (0.99) and French (0.96) versions of the MFES also indicated the high degree of repeatability in community-dwelling older people aged over 60 [14,24]. Additionally, Okuyan et al. founded that the test-retest correlation coefficient was significantly high for all items (r = 0.95) [23]. Therefore, we concluded that the internal consistency of the Turkish version of the MFES has high test-retest reliability (Table 2). These results show that the MFES is a useful scale to evaluate fall-related self-efficacy in repeated measures for elderly subjects from different countries.

In a recent study, Cronbach alpha values showed that the Turkish version of the MFES had high internal consistency and homogeneity. The Cronbach alpha value (0.978) obtained from the MFES2 was the same as that obtained from the MFES1. Our results were in parallel with the findings of different versions of the MFES, including Turkish (0.97) Serbian (0.98), Chinese (0.94), French (0.96), and the original version (0.95) [13,14,23–25]. The similarity between the Cronbach alpha values of the other studies in the literature and this study strengthens the reliability of our results. In addition, in relation to the Cronbach alpha if item deleted (CA-iid) values (0.975–0.978), we found that removing any item from the scale did not change the internal consistency (Table 2). Okuyan et al. stated that the analysis of the nonreverse principal components showed that the items of the measuring tool were loaded on two factors [23]. However, we found that the factor loadings obtained as a result of the principal component analysis indicated that the scale had a one-factor structure (78.28%) and all items could be used (Table 2).

Ulus et al. reported that fall-related self-efficacy worsened with increasing risk of falling [26]. Meanwhile, mobility impairments may cause disability progression in mobility as older adults fall or limit their movements after falling [23]. We found that the high-fall-risk group had lower MFES scores than the moderate- and low-fall-risk groups regarding the BBS scores (χ2 = 12.382, P = 0.002) (Table 4). O’Halloran et al. also stated that more attention must be paid to the other risk factors, such as increased extension of reaction times, variability in reaction time, and omission error rates [2]. Both previous and secondary fall-related factors have an important role in the risk of falling. When we investigated the differences between the fall-related self-efficacy of participants according to the demographic properties, we determined that the MFES scores significantly differed in terms of the mental and marital status, hearing problems, use of assistive devices, living environment, and history of falling (P < 0.05, Table 5). This study especially indicated that the degradation in mental status, according to the MMSE, contributed to a decrement in fall-related self-confidence, which was in parallel with the study conducted by Hauer et al. (P = 0.001) [12]. In relation with the use of assistive devices, participants obliged to use wheelchairs had lower MFES scores than the users of other assistive device (χ2 = 24.139, P = 0.001) (Table 5). No such comparison was made in the original MFES. Meanwhile, the previous studies pointed out that fall-related self-efficacy was associated with a history of falls and the use of assistive devices [11,14,27]. On the contrary, in the study of the Turkish version of FES-I, Ulus et al. defined that fall efficacy was associated with the use of assistive devices but not with the history of falls [26]. In this study, 71 of 164 participants had a history of falls and we found that their MFES scores were significantly lower than those without any history of falls (5.54 ± 2.11 and 6.84 ± 2.21, respectively) (P = 0.001, Table 5). Therefore, our findings have substantial clinical relevance for contributions regarding the fall-related self-efficacy among elderly individuals. Older adults with negative effects on fall-related self-efficacy are at a greater risk of indoor and outdoor activity restrictions and falls [24,28]. In this respect, Harding et al. reported that decline in self-efficacy gave an idea about changes in activities of daily living [20]. It should be noted that some demographics of elderly adults are important factors for the contribution of fall-related self-efficacy during different activities. Neupert et al. documented that exercising decreased personal barriers, including low self-efficacy and control of beliefs [29]. In this study, only 26 participants were exercising regularly and their MFES scores were not statistically different from those who did not exercise. This was an unexpected result, and it might have occurred because quite a low percentage of the elderly participants were exercising regularly and we did not question the type of exercise they did. We do not know whether these regular exercises were part of their lifestyle or an exercise protocol offered by a physiotherapist for their health problems. Hence, the type or aim of regular exercise could also be questioned in further studies focusing on fall-related self-efficacy. While the MFES scores were not different between the sexes (P > 0.05), there was a significant difference between the groups according to the living environment, especially among the participants who were living with a spouse and/or children (P = 0.006, Table 5), similar to the study of the original French version of the MFES. In addition to evidence comparing MFES scores of elderly individuals in terms of demographic properties for the good construct validity of the MFES, the results of correlation analysis obtained between MFES scores and history and frequency of falling (r = 0.292, P = 0.000 and r = –0.274, P = 0.000, respectively), and the BBS score (r = –0.465, P = 0.000), showed significant correlations despite the low/moderate correlation coefficients. Therefore, the MFES might have validity to predict balance problems. 

We interpreted these findings with the support of the literature and found that the Turkish version of the MFES is a sensitive and useful instrument for the evaluation of fall-related self-efficacy and in predicting falls, reduced physical activity, balance and mobility problems, and restricted social participation and activities of daily living.

This study has a few potential limitations. The participants of the study included older people who were living in different environments and situations, such as at their own homes alone or with family or in nursing homes. In addition, we were not able to determine the sensitivity of the Turkish version of the scale at various instances of time. Follow-up studies for 2–3 years within intervals of 6 months could help to obtain the predictivity and sensitivity of the MFES over time. The participants also primarily comprised older people with high functional levels in terms of physical, psychological, and cognitive capacities. Older people with cognitive impairment and/or specific medical conditions, such as stroke and Parkinson’s disease, could be included in the future studies.

The results of this study reveal that the Turkish version of the MFES is a reliable and valid scale to evaluate the fear of falling and self-confidence in elderly Turkish adults for both indoor and outdoor activities, and also in predicting falls and especially balance and mobility problems, reduced physical activity, and restricted social participation and activities of daily living. Therefore, with the use of the MFES, both researchers and clinicians could assess and follow the changes in fear of falls, in addition to formulating balance problems and fall prevention interventions, thereby improving the safety of the elderly while performing various balance-based daily activities in the community. Further studies to explore the application and responsiveness of the Turkish version of the MFES with changes over time in a wider spectrum of older people with different health conditions are needed. 

## Acknowledgements

The authors would like to thank Professor Hill for her permission to translate the MFES and for her advice on the research, as well as all participants and the centers that participated in this study. Special thanks to physiotherapist Ecem Yıldız Çensi, Gizem Töre, Tuğçe Bal, Şeyhmus Kazan, and Hanife Köse for their kind assistance in collecting the data from 60 of the 164 elderly participants. For the expenses of Ecem Yıldız Çensi, Gizem Töre, Tuğçe Bal, Şeyhmus Kazan, and Hanife Köse, a total of 2000 TL was received from TÜBİTAK-BIDEB - 2013/2 -1919B011303570. 

## Informed consent

Written consent was obtained from all of the participants.
